# Evidence that Processing of the Severe Fever with Thrombocytopenia Syndrome Virus Gn/Gc Polyprotein Is Critical for Viral Infectivity and Requires an Internal Gc Signal Peptide

**DOI:** 10.1371/journal.pone.0166013

**Published:** 2016-11-17

**Authors:** Teresa Plegge, Heike Hofmann-Winkler, Martin Spiegel, Stefan Pöhlmann

**Affiliations:** 1 Abteilung Infektionsbiologie, Deutsches Primatenzentrum, Göttingen, Germany; 2 Institut für Mikrobiologie und Virologie, Medizinische Hochschule Brandenburg Theodor Fontane, Senftenberg, Germany; University of Minnesota College of Veterinary Medicine, UNITED STATES

## Abstract

The severe fever with thrombocytopenia syndrome virus (SFTSV) is an emerging, highly pathogenic bunyavirus against which neither antivirals nor vaccines are available. The SFTSV glycoproteins, Gn and Gc, facilitate viral entry into host cells. Gn and Gc are generated from a precursor protein, Gn/Gc, but it is currently unknown how the precursor is converted into the single proteins and whether this process is required for viral infectivity. Employing a rhabdoviral pseudotyping system, we demonstrate that a predicted signal sequence at the N-terminus of Gc is required for Gn/Gc processing and viral infectivity while potential proprotein convertase cleavage sites in Gc are dispensable. Moreover, we show that expression of Gn or Gc alone is not sufficient for host cell entry while particles bearing both proteins are infectious, and we provide evidence that Gn facilitates Golgi transport and virion incorporation of Gc. Collectively, these results suggest that signal peptidase liberates mature Gc from the Gn/Gc precursor and that this process is essential for viral infectivity and thus constitutes a potential target for antiviral intervention.

## Introduction

Bunyaviruses constitute the largest RNA virus family and infect a wide range of hosts, including humans, arthropods and plants. Several emerging bunyaviruses pose a considerable threat to human health as exemplified by Rift Valley fever virus (RVFV) [1], Crimean Congo hemorrhagic fever virus (CCHFV) [2] and severe fever with thrombocytopenia syndrome virus (SFTSV) [3,4], which can cause severe disease in afflicted patients. SFTSV, a novel member of the phlebovirus genus, emerged in 2007 in Central and Eastern China [5,6]. The virus is transmitted from ticks to humans, with human to human transmission occurring on rare instances [7–9], and can induce a severe disease characterized by fever, gastrointestinal symptoms and thrombocytopenia. The case-fatality rate is approximately 10% in China, with the elderly being disproportionately affected, but higher rates have been observed upon SFTSV outbreaks in South Korea [10] and Japan [11]. Moreover, a virus closely related to SFTSV, Heartland virus, has been identified in the US and infection was associated with severe disease [12]. Thus, SFTSV and related viruses are important emerging agents against which at present neither specific antivirals nor vaccines are available.

Bunyaviruses contain a tripartite RNA genome consisting of three single stranded RNA segments of negative polarity termed L, M and S. The M segment encodes for the viral envelope proteins Gn and Gc, which facilitate viral entry into target cells and are the major targets for neutralizing antibodies [13,14]. In addition, the M segments of some bunyaviruses encode a non-structural protein (NSm) but no NSm open reading frame was identified in the M segment of SFTSV. The Gn and Gc proteins of SFTSV have been shown to mediate entry into several cell lines as well as primary dendritic cells and macrophages [15,16]. Entry into dendritic cells depends on the cellular lectin DC-SIGN [15], which can interact with pathogens in a glycan-dependent manner. Moreover, Gn binds to non-muscle myosin heavy chain IIA and expression of this protein is required for efficient SFTSV infection of cell lines [17]. Thus, SFTSV employs the Gn and Gc proteins as keys for entry into target cells and elucidating the processing and function of these proteins might reveal targets for antiviral intervention.

Bunyavirus Gn and Gc are generated from a precursor polyprotein, frequently termed Gn/Gc, which is proteolytically processed into the single Gn and Gc proteins and, in case of CCHFV and several other bunyaviruses, into additional viral glycoproteins and a non-structural protein, NSm [18]. For some bunyaviruses, a role of signal peptidase in Gn/Gc processing has been reported [19–21]. In addition, the activity of a proprotein convertase, subtilisin/kexin-isozyme 1 (SKI-1, also known as site-1 protease (S1P)), was shown to be required for conversion of a precursor form of CCHFV Gn into the mature Gn protein [22,23]. How the SFTSV Gn/Gc precursor is converted into Gn and Gc proteins and whether this process is required for viral infectivity is at present unclear.

Here, we show that processing of the Gn/Gc precursor into Gn and Gc is a prerequisite to infectious viral entry and we provide evidence that Gn promotes virion incorporation of Gc. Moreover, we demonstrate that potential proprotein convertase cleavage sites in Gc are dispensable for processing of the Gn/Gc precursor, while the integrity of a predicted signal sequence at the N-terminus of Gc is essential. These findings indicate that the cellular enzyme signal peptidase liberates Gc from the Gn/Gc precursor and that this process is required for viral infectivity.

## Materials and Methods

### Cell culture

Human embryonic kidney 293T cells (ATCC CRL-3216) and African green monkey COS-7 (ATCC CRL-1651) kidney cells were maintained in Dulbecco’s modified Eagle’s medium (DMEM; PAN Biotech) supplemented with 10% fetal bovine serum (FBS; Biochrome), 100 U/ml penicillin and 100 μg/ml streptomycin (PAN Biotech), and 1% L-glutamine (PAN Biotech). The cells were grown in a humidified atmosphere at 37°C and 5% CO_2_. The cells were obtained from collaborators and their identity was confirmed by STR DNA typing employing a published protocol [24].

### Plasmids

Expression plasmids encoding severe fever and thrombocytopenia syndrome virus glycoprotein (SFTSV-Gn/Gc) [15], Rift valley fever virus glycoprotein (RVFV-Gn/Gc) [15], La Crosse virus glycoprotein (LACV-Gn/Gc) [15], Lassa virus glycoprotein (LASV-GPC) [25], Ebola virus GP (EBOV-GP) and Middle East respiratory syndrome coronavirus spike protein (MERS-S) with and without C-terminal V5 tag [26] have been described previously. A plasmid encoding the vesicular stomatitis virus glycoprotein (VSV-G) has also been described previously [25]. The SFTSV-Gn/Gc-V5 sequence inserted into plasmid pCAGGS served as basis for the generation of further constructs. Expression plasmids for SFTSV-Gn (aa 1–543) and SFTSV-Gc (aa 536–1073) with and without C-terminal V5- or myc-tag were generated by polymerase chain reaction (PCR) and cloning of PCR products into plasmid pCAGGS using Asp718 and XhoI restriction sites. The start codon of Gc within the Gn/Gc precursor sequence was mutated by PCR using the primers 5‘-GATCGGTACCACCATGATGAAAGT GATCTGGTTCAGCAGCC-3‘ and 5‘-CAGTGCCGCACGCGTCTAGGTCTGGCTGCCTCGATCCG-3‘ (CAT to TGC mutation underlined). The PCR product was inserted into plasmid pCAGGS using Asp718 and MluI restriction sites, resulting in mutant mutATG-Gc. To mutate RRxR motifs within Gc, overlap extension PCR was employed. Specifically, primers 5‘-CCAGTGCACCACGGCTGCAGGTCTGGCCATCTCGATCC-3‘ and 5‘-GCCAGACCTGCAGCCGTGGTGCACTGGATGTACAGCCCAGTGATCC-3‘ were used as inner primers to generate mutant mutRRxR-1 (540-AAVV-543) while 5‘-CCAGCGTGGCGGCATGTGCATGGGCAGGCGATTGCCAGAGCGG-3‘ and 5‘-TGCACATGCCGCCACGCTGGTACATCTGGACCGGGCGTCGGGC-3‘ served as inner primers for generation of mutant mutRRxR-2 (648-AACA-651). To generate mutant mutATG-Gc-RRxR-1, the inner primers 5‘-GCCAGACCTGCAGCCGTGGTGCACTGGATGTACAGCCCAGTGATCC-3’ and 5‘-CCAGTGCACCACGGCTGCAGGTCTGGCTGCCTCGATCCGCACG-3‘ were used. The PCR product encoding mutRRxR-2 was directly inserted into pCAGGS using MluI and XhoI restriction sites. In contrast, the PCR products encoding mutRRxR-1 and mutATG-Gc-RRxR-1 were first inserted into pcDNA3.1zeo containing a partial SFTSV fragment followed by insertion of the entire coding sequence into pCAGGS via Asp718 and XhoI restriction sites. The PCR and cloning strategy used for generation of mutants mutRRxR-1 and mutATG-Gc-RRxR-1 was also employed to generate mutants ΔSigP-V5 (Δ536–560) and ΔLAIGLAEG-V5 (Δ555–562). Specifically, primers 5‘-GGCAACCAGGACGACGTGCGGATCGAG GGCTGTGACGAAATGGTGCACGCCGAC-3’ and 5‘-GTCGGCGTGCACCATTTCGTCACAGCCCTCGATCCGCACGTCGTCCTGGTTGCC-3‘ were used as inner primers to generate SFTSVΔSigP-V5 (Δ536–560) while inner primers 5’-CCAGTGATCCTGACCATCTGTGACGAAATGGTGCAC-3’ and 5’-GTGCACCATTTCGTCACAGATGGTCAGGATCACTGG-3’ were employed for constructing SFTSVΔLAIGLAEG-V5 (Δ555–562). The integrity of all PCR-amplified sequences was confirmed by in house (German Primate Center) automated sequence analysis (Sanger method). Plasmids pE-GFP-ER, which encodes calreticulin fused to GFP [27], and pE-GFP-Golgi, which encodes beta-1,4-galactosyltransferase fused to GFP [28], were used to visualize the endoplasmic reticulum and the Golgi apparatus, respectively.

### Analysis of glycoprotein expression and cleavage

293T cells were seeded in 6-well plates at 250,000 cells per well, incubated for 24 h and then calcium-phosphate-transfected with 2 μg of the respective glycoprotein expression plasmids. After incubation for 6 h, the cells were washed twice with warm phosphate buffered saline (PBS) and new culture medium was added. At 48 h post transfection, cells were harvested and lysed in 2 x sodium dodecyl sulfate (SDS)-lysis buffer (1.5 ml 1M Tris pH 6.8, 6 ml 80% Glycerol, 10 ml 10 x SDS, 2.5 ml β-mercaptoethanol, 5 ml 1% bromphenol blue, 50 μl 1M EDTA). For analysis of N-glycosylation, PNGase F digest was employed, using commercially available reagents (New England BioLabs, Ipswich, MA, USA). For this, transfected cells were resuspended in denaturing buffer, incubated at 100°C for 10 minutes and then exposed to 500 units PNGase F at 37°C for 1 hour.

For Western blot analysis, cell lysates were separated by SDS polyacrylamide gel electrophoresis (PAGE) and blotted onto nitrocellulose membranes (GE Healthcare, Life Sciences, Freiburg, Germany). Prior to antibody staining, membranes were blocked with 5% milk in PBS with 0.1% Tween. Expression of viral glycoproteins was analyzed with mouse monoclonal antibodies directed against the V5 tag (Invitrogen, Karlsruhe, Germany) or the myc tag [29]. As loading control, β-actin expression was detected using a mouse monoclonal antibody (Sigma-Aldrich, Taufkirchen, Germany). A horseradish peroxidase-(HRP)-conjugated secondary antibody (Dianova, Hamburg, Germany) and a chemiluminescence-based commercially available kit (GE Healthcare Life Sciences, Freiburg, Germany) were used for visualization of bound antibodies. Signals were detected with Intas ChemoCam Image 3.2. Quantification of signal intensities was performed using Fiji/ImageJ 1.51g software.

### Inhibition of glycoprotein cleavage

For inhibition of cellular proteases, the following compounds were used: Proprotein convertase inhibitor (Merck Millipore, Darmstadt, Germany), AEBSF (4-(2-aminoethyl)-benzenesulfonylfluoride hydrochloride) and PF-429242 dihydrochloride (4-[(Diethylamino)methyl]-N-[2-(2-methoxyphenyl)ethyl]-N-(3R)-3-pyrrolidinyl-benzamide dihydrochloride) (both Sigma-Aldrich, Taufkirchen, Germany). All inhibitors were diluted in solvent as recommended by the manufacturers and applied in the indicated concentrations. Target cells were treated with the inhibitors from 6 h post transfection until cells were harvested.

### Analysis of SFTSV-Gn/Gc-driven host cell entry

For analysis of viral entry a previously described rhabdoviral pseudotyping system was used [15]. In brief, 293T cells were seeded in T-25 cell culture flasks at 500,000 cells/flask and calcium-phosphate-transfected with 6 μg of the glycoprotein expression plasmid. After a 6 h incubation period, the cells were washed twice with warm PBS and fresh culture medium was added. At 30 h post transfection, the cells were transduced at an MOI of 0.2 with a replication-defective vesicular stomatitis virus (VSVΔG) pseudotyped with VSV-G and encoding luciferase and green fluorescent protein (GFP). After a 1 h incubation step, the cells were washed five times with PBS and fresh culture medium was added. Twenty-four hours post transduction rhabdoviral pseudotypes were harvested from the culture supernatants via sterile-filtration through 0.45 μm filters and stored at -80°C. For analysis of infectivity, 293T cells were seeded in 96-well plates at 30,000 cells/well. At 24 h post seeding, cells were incubated with 50 μl pseudotyped virus for 1 h followed by replacement of the medium by fresh culture medium. Transduction efficiency was quantified at 24 h post inoculation by determining luciferase activity in cell lysates using a commercially available beetle-juice luciferase assay kit (p.j.k., Kleinblittersdorf, Germany). For analysis of particle incorporation of viral glycoproteins, sterile-filtered virus stocks were concentrated by centrifugation through a 20% sucrose cushion (17,000 g, 2 h), lysed and analyzed by SDS-PAGE and Western blotting, as described above. A monoclonal anti-VSV-M antibody (Kerafast, Boston, MA, USA) was used to detect the presence of M-protein in viral particles.

### Immunofluorescence and confocal microscopy

COS-7 cells were seeded on glass coverslips in 24-well plates at 40,000 cells per well and incubated for 24 h at 37°C. Subsequently, the cells were calcium-phosphate cotransfected with 0.5 μg of plasmids encoding viral glycoproteins (SFTSV-Gn-myc and/or SFTSV-Gc-V5 or SFTSV-Gn/Gc-V5) and marker proteins localized to the endoplasmic reticulum (calreticulin fused to GFP, [27]) or Golgi apparatus (β-1,4-galactosyltransferase fused to GFP, [28]). Transfection of empty pCAGGS plasmid served as negative control. After 6 h of incubation at 37°C, cells were washed twice with PBS and fresh culture medium was added. At 48 h post transfection, the glass coverslips were washed once with PBS followed by fixation in 4% paraformaldehyde for 10 min at RT. After washing three times with PBS, the glass coverslips were incubated in 0.2% Triton X-100 in PBS for 10 min at RT and washed three times with PBS. Prior to antibody staining, samples were blocked for 30 min at 37°C with PBS containing 0.5% Tween and 1% bovine serum albumin (BSA). The primary antibodies mouse monoclonal IgG2a anti-V5 (Invitrogen, Karlsruhe, Germany) and rabbit polyclonal anti-c-Myc (Sigma-Aldrich, Taufkirchen, Germany) were diluted 1:500 in PBS containing 0.5% Tween and 1% BSA and glass coverslips were placed upside down on 25 μl droplets of the antibody dilutions and incubated at RT for 1 h. Glass coverslips were washed three times with PBS and again incubated upside down on 25 μL droplets containing secondary antibodies, Alexa fluor 647 donkey anti-mouse IgG (Life Technologies, Eugene, OR, USA) and Alexa fluor 546 donkey anti-rabbit IgG (Life Technologies, Eugene, OR, USA) diluted 1:1,000 in PBS supplemented with 0.5% Tween and 1% BSA in PBS. After 1 h incubation at RT in the dark, the glass coverslips were washed three times with PBS followed by two washes with distilled H_2_O prior to mounting onto a microscope slide with mowiol. Fluorescence images were acquired with the confocal laser scanning microscope LSM 5 Pascal Axioskop 2 MOT plus (Carl Zeiss Microimaging GmbH, Göttingen, Germany) using a 63x, NA1.4 Zeiss Plan Apochromat objective and LSM Pascal 5 software version 3. Confocal images were colored and converted to JPEG using the software AxioVision Rel. 4.8 (Carl Zeiss Microimaging GmbH). All images were cropped to 70% of the original image for presentation purposes. No further image modifications were performed.

### Statistical analysis

Statistical analysis was performed by Welch’s t-test [30].

## Results

### The SFTSV-Gn/Gc precursor is efficiently processed into Gn and Gc

We first asked whether expression of the SFTSV-Gn/Gc precursor protein results in production of mature Gn and Gc proteins in 293T cells, a highly transfectable cell line previously used to generate infectious SFTSV-Gn/Gc-bearing vectors [15]. For this, cells were transfected with plasmid encoding the Gn/Gc precursor protein of SFTSV. For comparison, Gn/Gc of RVFV and LACV, other members of the bunyavirus family, were also studied. In addition, the glycoproteins of Ebola virus (EBOV-GP) and Lassa virus (LASV-GPC) were analyzed as positive controls for glycoprotein processing, since they are known to be cleaved by proprotein convertases [31,32]. All glycoproteins studied contained a C-terminal V5 antigenic tag for convenient detection by Western blot.

**Fig 1:**
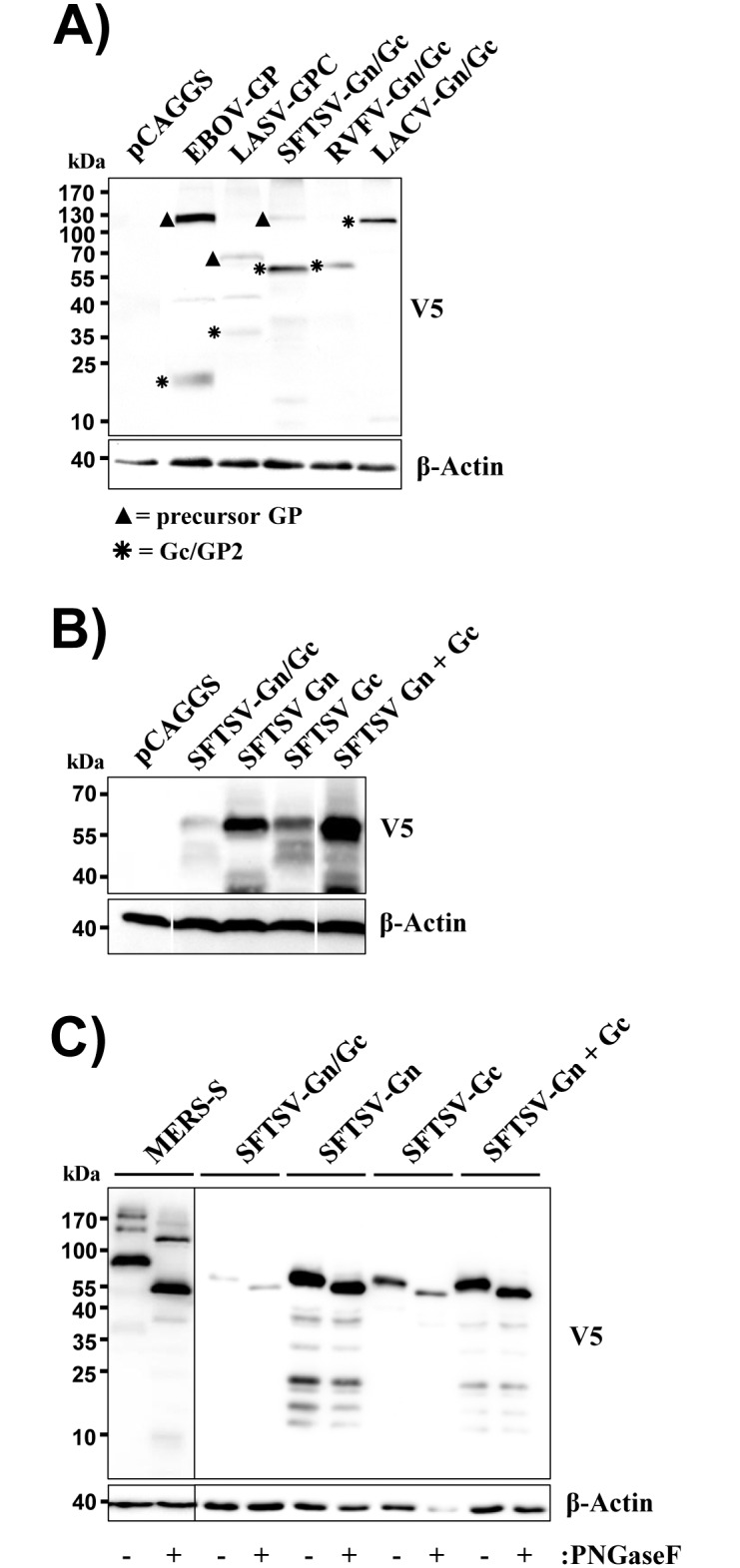
Gn and Gc proteins are efficiently expressed from separate plasmids. (A) 293T cells were transfected with empty pCAGGS plasmid or pCAGGS encoding the indicated viral glycoproteins with a C-terminal V5 antigenic tag. Glycoprotein expression was analyzed by Western blotting using a V5-specific monoclonal antibody (top panel). Detection of β-actin served as loading control (bottom). Unprocessed glycoprotein precursor proteins are marked with filled triangles while asterisks indicate processed Gc (SFTSV, RVFV, LACV) or GP2 (EBOV, LASV). A single representative blot is shown from which irrelevant lanes were excised. Similar results were obtained in three independent experiments. (B) The experiment was conducted as described for panel A but plasmids encoding both the Gn/Gc precursor and single proteins (all containing a V5 tag) were transfected. A single representative blot is shown from which irrelevant lanes were excised. Similar results were obtained in three independent experiments. (C) The experiment was conducted as described for panel B but cell lysates were treated with PNGaseF to remove N-glycans or Mock treated. As a control for successful removal of N-glycans, cells expressing the spike protein of MERS-CoV were PNGase F treated. The results were confirmed in three independent experiments.

For EBOV-GP and LASV-GPC, the expected C-terminal processing products of 20 kDa and 35 kDa were detected in addition to the precursor proteins (Fig 1A), indicating that these glycoproteins were processed by host cell proteases. Similarly, signals corresponding to the molecular weight expected for mature Gc were detected in cells expressing SFTSV, RVFV and LACV Gn/Gc proteins (Fig 1A). In addition, a weak signal of the molecular weight expected for the SFTSV-Gn/Gc precursor (116 kDa) was detected. These results indicate that SFTSV-Gn/Gc and other bunyavirus Gn/Gc precursor proteins were efficiently processed into mature proteins under the conditions studied.

### SFTSV-Gn and Gc are both required for infectious entry

We next asked whether Gn and Gc proteins can be expressed separately and whether expression of both proteins is required for host cell entry. Mature Gn and Gc are predicted to exhibit almost identical molecular weights of 60 kDa and 58 kDa, respectively, and the corresponding bands were indeed detected in cells transfected with separate expression plasmids encoding these proteins (Fig 1B). For at present unclear reasons, expression of Gc from a plasmid encoding only the Gc ORF was more efficient than expression from a Gn/Gc encoding plasmid, and Gn was expressed to higher levels than Gc (Fig 1B). Digestion with PNGase F revealed that Gn and Gc expressed from separate plasmids were N-glycosylated (Gn harbors two consensus sequences for N-glycosylation while three consensus sequences are present in Gc), indicating that these proteins enter the secretory pathway (Fig 1C), as expected.

**Fig 2:**
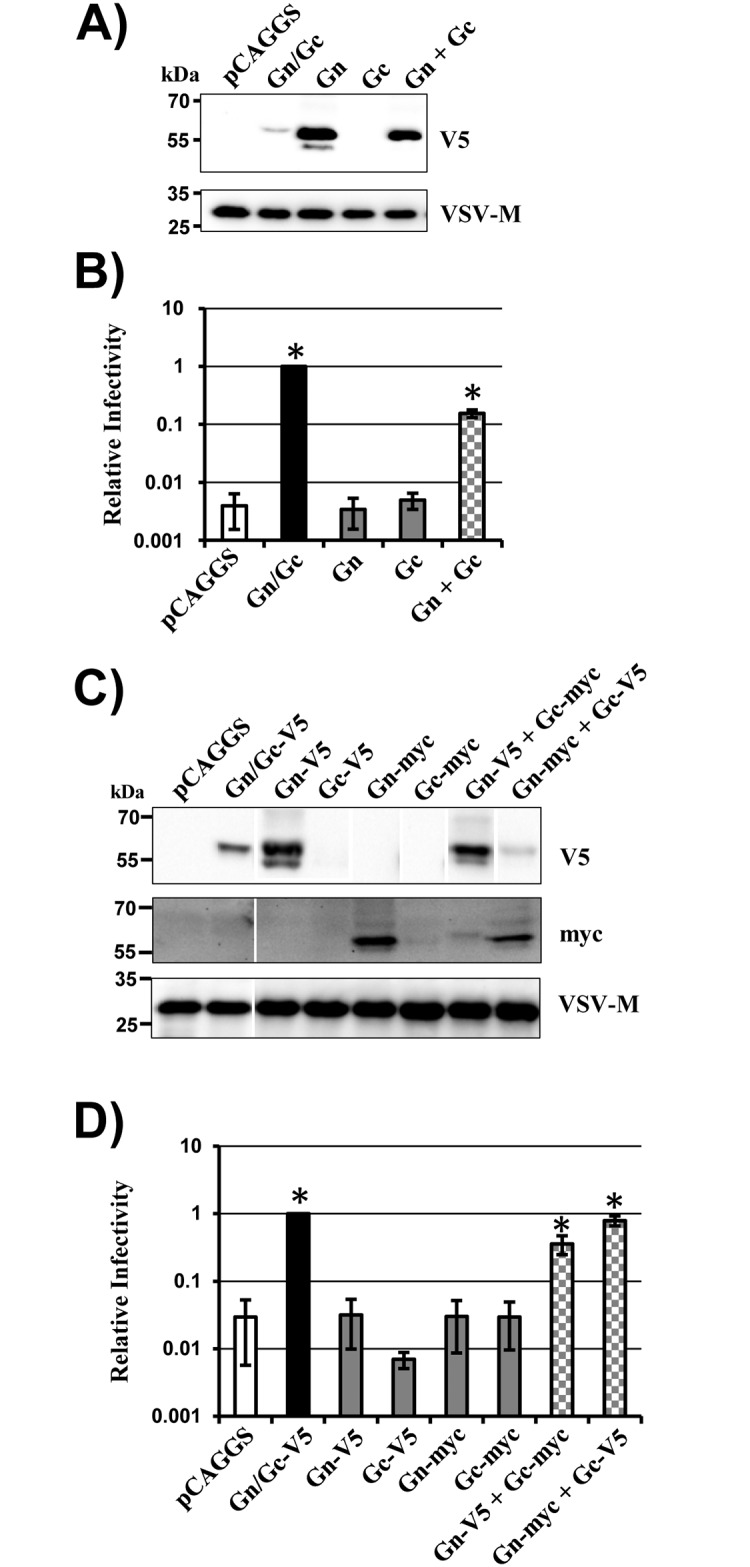
Gn facilitates virion incorporation of Gc and both proteins are required for host cell entry. (A-B) Rhabdoviral vectors encoding luciferase and pseudotyped with the indicated glycoproteins with V5 tag were analyzed for incorporation of glycoprotein and VSV matrix (M) protein (panel A) and for transduction of 293T cells (panel B). Transduction efficiency was analyzed by determining luciferase activities in lysates of transduced cells. Transduction measured with particles generated in Gn/Gc expressing cells was set as 1. Particles harboring no glycoprotein (pCAGGS) were used as a control. (C-D) The experiment was carried out as described for panels A-B but the indicated V5- and myc-tagged glycoproteins were used. For infectivity assays (panels B and D), the average of three independent experiments performed with triplicate samples are shown. Error bars indicate standard error of the mean (SEM). Asterisks indicate a statistical significant increase (p<0.05) of relative infectivity with respect to the control (pCAGGS). The virion incorporation data were confirmed in at least four independent experiments. Representative blots are shown in panel C from which irrelevant lanes were excised.

Based on our finding that separate expression of Gn and Gc is feasible, we next asked whether both proteins are required for infectious entry. For this, we employed a vesicular stomatitis virus (VSV)-based pseudotyping system previously shown to be suitable for analysis of SFTSV-Gn/Gc-driven entry [15]. Particles produced in cells transfected with Gn/Gc encoding plasmid incorporated mature Gc (we did not have the means to analyse Gn incorporation) and were readily able to transduce cells (Fig 2A and 2B), although with roughly 100-fold reduced efficiency compared to VSV-G bearing particles, in line with our previous findings [15]. In contrast, expression of Gn alone was not sufficient to render particles infectious, despite efficient particle incorporation of the protein (Fig 2A and 2B). Moreover, particles produced in the presence of Gc did not harbor Gc and were not infectious. Finally, particles produced in cells cotransfected with separate Gn and Gc plasmids were able to transduce cells, although with slightly reduced efficiency as compared to particles from Gn/Gc expressing cells (Fig 2A and 2B). These results suggest that both Gn and Gc are required for infectious SFTSV entry and demonstrate that simultaneous expression of these proteins from separate plasmids is compatible with infectivity.

### Gn is required for particle incorporation of Gc

Mature Gc was incorporated into particles upon expression of the Gn/Gc precursor protein but not upon expression of Gc alone, as discussed above. These findings posed the question whether Gn facilitates virion incorporation of Gc. To investigate this possibility, particle incorporation of the viral glycoproteins and infectivity were analyzed upon coexpression of vector with combinations of Gn and Gc proteins harboring either the V5 or the myc antigenic tag. These studies confirmed that expression of Gc alone is not compatible with particle incorporation and infectivity (Fig 2C and 2D). In addition, they demonstrated that Gc is incorporated into particles upon coexpression of Gn and showed that simultaneous particle incorporation of both Gn and Gc is associated with infectivity (Fig 2C and 2D).

### Gn facilitates transport of Gc from the endoplasmic reticulum into the Golgi apparatus

**Fig 3:**
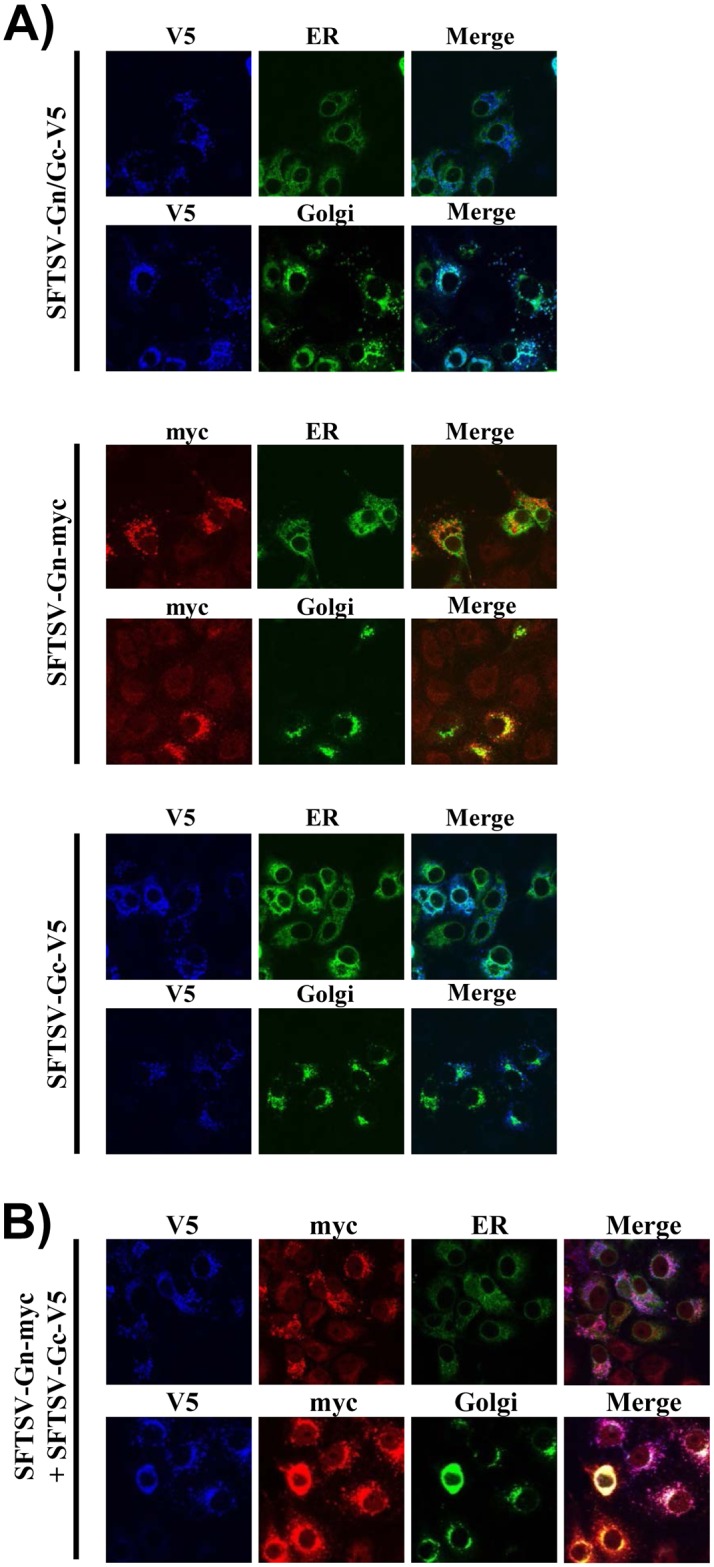
Gn facilitates Gc transport into the Golgi apparatus. COS-7 cells seeded on glass coverslips were cotransfected with plasmids encoding the indicated glycoproteins (panel A: SFTSV-Gn-myc, SFTSV-Gc-V5 or SFTSV-Gn/Gc-V5; panel B: SFTSV-Gn-myc and SFTSV-Gc-V5) and markers for the endoplasmic reticulum and the Golgi apparatus, respectively. At 48 h post transfection, immunofluorescence staining was performed using antibodies directed against the antigenic tags as primary antibodies and Alexa fluor 647- and Alexa fluor 546-labelled antibodies as secondary antibodies. Fluorescence images were taken at 63x resolution and cropped to 70% of the original image for presentation purposes. No further image modifications were performed. Similar results were obtained in a separate experiment.

The finding that Gn is required for virion incorporation of Gc raised the question whether Gn might facilitate the transport of Gc to the site of viral budding, the Golgi apparatus. To address this question, we analyzed the cellular localization of epitope tagged Gn, Gc and Gn/Gc via confocal laser scanning microscopy. Cellular proteins fused to GFP and known to localize to the endoplasmic reticulum (calreticulin) and the Golgi apparatus (β-1,4-galactosyltransferase), respectively, were employed as organelle markers. We found that Gn and Gn/Gc were predominantly localized to the Golgi apparatus while Gc alone was mainly found in the ER (Fig 3A). Moreover, coexpression of Gn with Gc resulted in accumulation of Gc in the Golgi apparatus (Fig 3B), indicating that Gn facilitates transport of Gc into this organelle.

### RRxR motifs in Gc are dispensable for Gn/Gc processing

**Fig 4:**
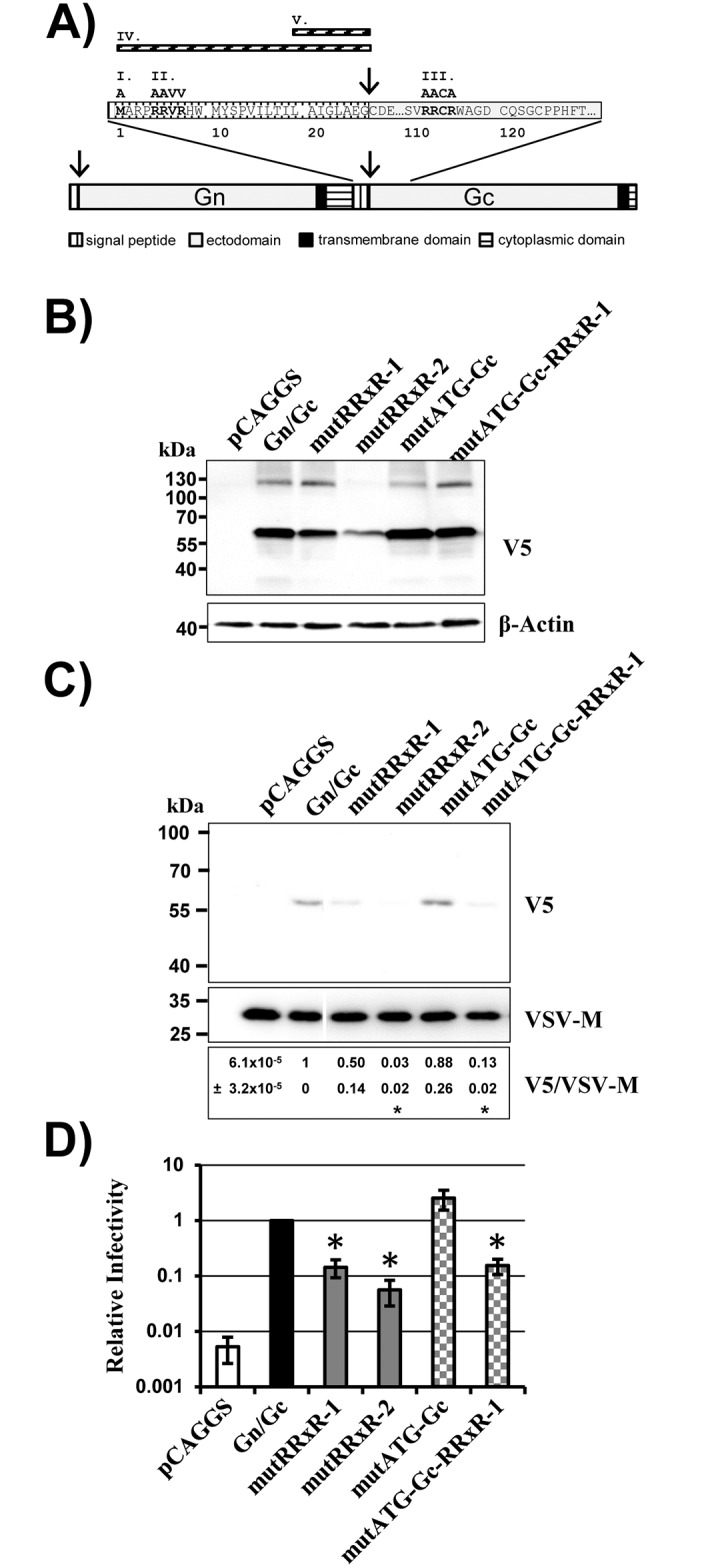
RRxR motifs and methionine at position 1 within Gc are dispensable for Gn/Gc processing. (A) Schematic overview of the SFTSV-Gn/Gc glycoprotein. Domains within Gn and Gc are indicated, the sequences of the putative signal peptide of Gc and internal sequences in Gc surrounding a RRxR motif are shown. Mutations introduced into the Gn/Gc precursor are shown in roman numerals. I., mutant mutATG-Gc, in which the methionine at position 1 was changed to an alanine. II. and III, mutants mutRRxR-1 and mutRRxR-2, in which RRxR motifs within the Gc signal peptide and in the Gc ectodomain were mutated as indicated. IV, mutant ΔSigP, in which the signal peptide of Gc was deleted. V. ΔLAIGLAEG, deletion of the eight amino acids upstream of the internal signal peptidase cleavage site. Numbering of the sequence cutout starts at the first amino acid of the internal Gc signal peptide. (B) 293T cells were transfected with empty plasmid (pCAGGS) or pCAGGS encoding the indicated glycoproteins. Glycoprotein expression was analyzed by Western blotting using a V5-specific antibody (top) or a β-actin antibody (bottom) as loading control. Similar results were obtained in at least three independent experiments. (C) Equal volumes of rhabdoviral vectors pseudotyped with SFTSV Gn/Gc wt or the indicated Gn/Gc mutants were pelleted through a sucrose cushion and pellets were analyzed by Western blot for incorporation of glycoproteins and matrix protein M. A single representative blot is shown from which irrelevant lanes were excised. The results were confirmed in three independent experiments and relative quantification of signal intensities was performed. Numbers represent the mean values of relative V5/VSV-M signal intensity ratios ± SEM. Asterisks denote a statistically significant decrease (p<0.05) of V5/VSV-M ratios with respect to the V5/VSV-M ratio of Gn/Gc wt. (D) The rhabdoviral pseudotypes analyzed in panel C were used for transduction of 293T cells. The average of three independent experiments performed with triplicate samples is shown. Transduction measured with particles generated in Gn/Gc expressing cells was set as 1. Error bars indicate SEM. Asterisks denote a statistical significant decrease (p<0.05) of relative infectivity with respect to particles bearing Gn/Gc.

We next asked which protease is responsible for Gn/Gc processing into mature Gn and Gc. In silico analyses predicted signal peptides at the N-termini of Gn and Gc (Fig 4A). Moreover, an RRxR motif, a potential cleavage site for proprotein convertases or related enzymes, was detected at position 5 within Gc (Fig 4A). Cleavage at this motif would be compatible with the observed molecular weight of Gc (Fig 1B). Therefore, we determined whether mutation of this motif impacts Gn/Gc cleavage (mutant RRxR-1). Mutation of an internal RRxR motif (position 113) within Gc served as control (mutant RRxR-2). Moreover, we addressed whether the N-terminal methionine of Gc is required for Gc expression (mutant mutATG-Gc), which would be expected if Gc was translated separately from Gn, potentially due to an unidentified IRES element. Finally, the effect of combined mutation of the first RRxR motif and the AUG was studied (mutant mutATG-Gc-RRxR-1).

**Fig 5:**
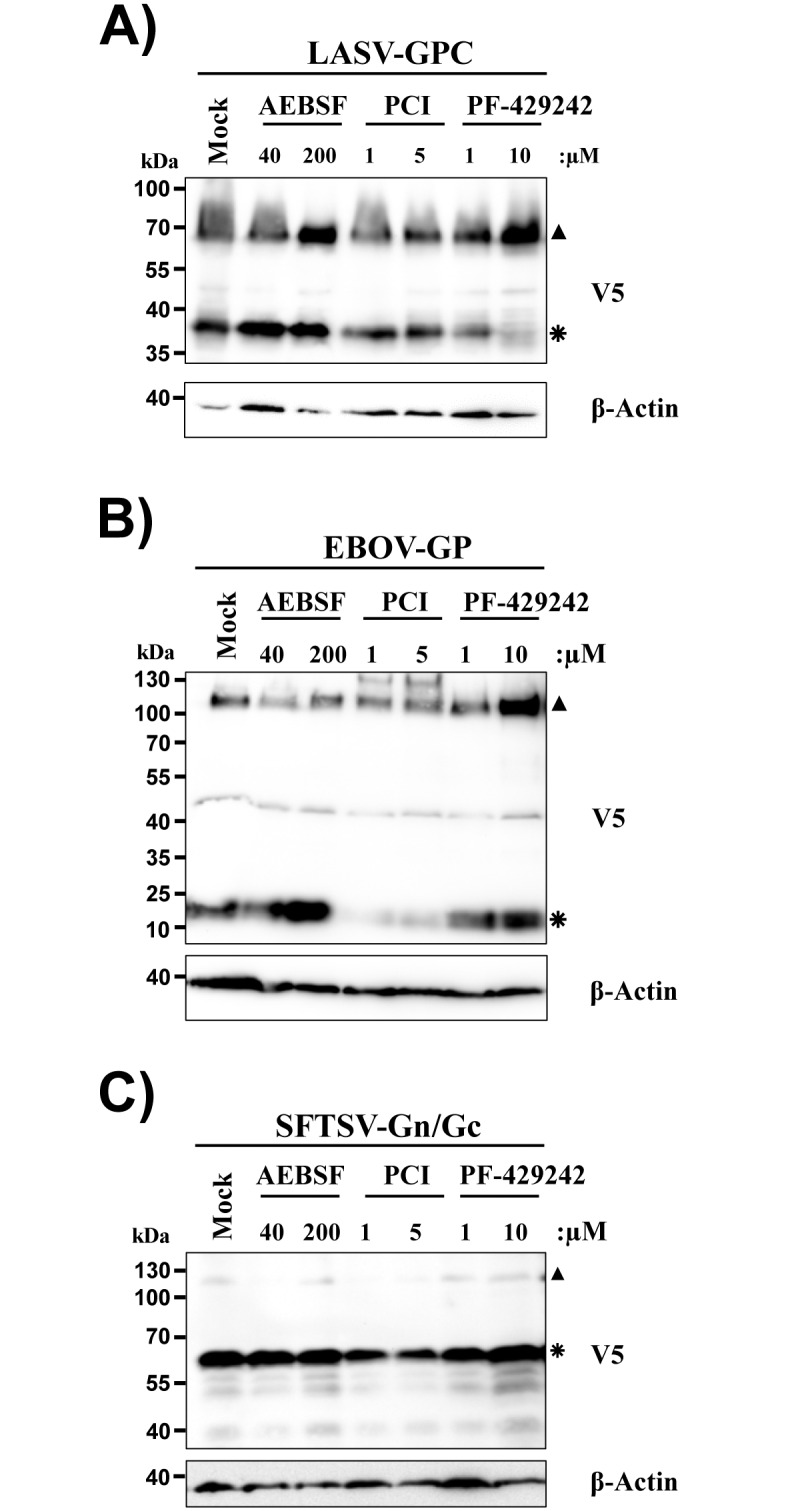
Proprotein convertase activity is dispensable for processing of SFTSV Gn/Gc. 293T cells were transfected with plasmid pCAGGS encoding SFTSV-Gn/Gc, EBOV-GP or LASV-GPC. At 6 h post transfection the indicated concentrations of the furin inhibitor PCI, the serine protease inhibitor AEBSF and the SKI-1 inhibitor PF-429242 were added or the cells were mock treated. Cells were lysed 48 h post transfection and cell lysates were analyzed for LASV-GPC (panel A), EBOV-GP (panel B) or SFTSV-Gn/Gc (panel C) expression by Western blotting using a V5-specific antibody. Expression of β-actin was determined as loading control. Unprocessed glycoprotein precursor proteins are marked with filled triangles while asterisks indicate processing products. Similar results were obtained in at least three independent experiments.

None of the mutations tested interfered with processing of the Gn/Gc precursor protein (Fig 4B), although mutation of the second RRxR motif reduced protein expression (Fig 4B). Moreover, mutation of either of two RRxR motifs reduced virion incorporation (Fig 4C) of Gc and particle infectivity (Fig 4D). In contrast, mutation of the ATG did not interfere with expression and virion incorporation of Gc or with particle infectivity (Fig 4C and 4D). These results suggest that the RRxR motifs in Gc do not constitute cleavage sites for proprotein convertases or other cellular proteases. Indeed, blockade of proprotein convertases inhibited processing of the LASV-GPC and EBOV-GP (Fig 5A and 5B), as expected [31,32], but had no impact on processing of SFTSV-Gn/Gc (Fig 5C). Similarly, blockade of a serine protease activity previously shown to be required in target cells for efficient Gn/Gc-driven entry [15] was dispensable for Gn/Gc processing (Fig 5). Collectively, our observations indicate that Gn/Gc processing is independent of proprotein convertase activity and RRxR motifs in Gc. Moreover, they suggest that the ATG of the Gc ORF does not serve as a start codon for translation in the context of the Gn/Gc mRNA.

### The predicted signal peptide in Gc is essential for Gn/Gc processing

**Fig 6:**
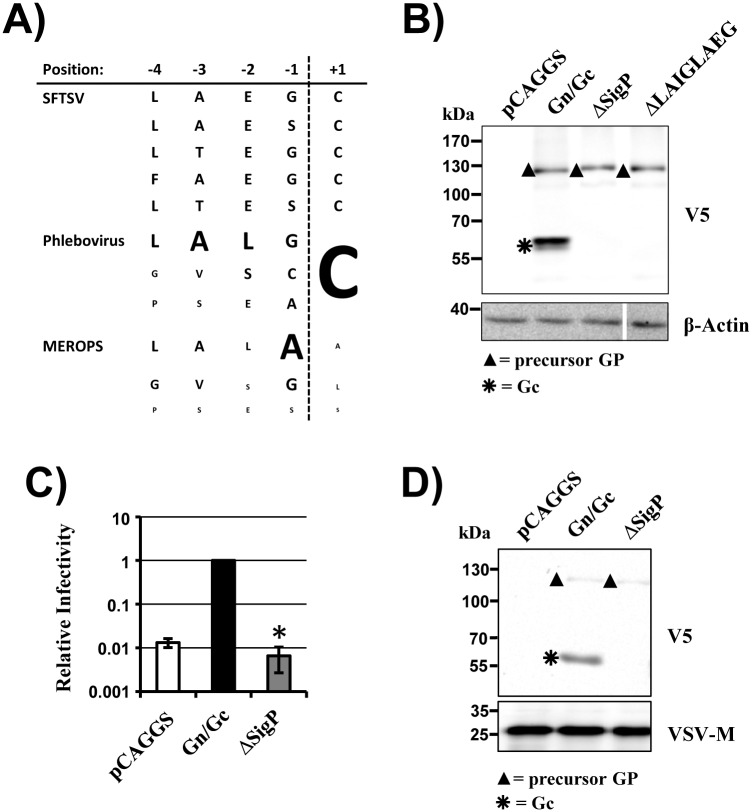
Deletion of the Gc signal peptide abrogates Gn/Gc processing and Gn/Gc-driven entry. (A) Predicted signal peptidase recognition and cleavage sites found in SFTSV-Gc variants (derived from Pubmed Acc. No. BAQ59274, AFK08659, JX462459, JX462462, KC392317). Depicted are the amino acid residues before (-4 to -1) and after (+1) the putative signal peptidase cleavage site. In comparison, the three most frequent amino acid residues for each position found in 103 phlebovirus Gn/Gc sequences and in the 1878 signal peptidase cleavage sites stored in the MEROPS database (http://merops.sanger.ac.uk/cgi-bin/pepsum?id=XS26-001;type=P) are shown. For the phlebovirus and MEROPS sequences the size of the letters corresponds to the frequency in percent of the respective amino acid found in this position. (B) 293T cells were transfected with empty plasmid (pCAGGS) or pCAGGS encoding SFTSV Gn/Gc wt or Gn/Gc with signal peptide deletions. Glycoprotein expression in cell lysates was analyzed by Western blotting using a V5-specific antibody (top) or a β-actin antibody (bottom). A single representative blot is shown from which irrelevant lanes were excised. Similar results were obtained in at least three independent experiments. (C) Rhabdoviral pseudotypes harboring the glycoproteins analyzed in panel A were used for transduction of 293T cells. The average of four independent experiments performed with triplicate samples is shown. Transduction measured with particles generated in Gn/Gc expressing cells was set as 1. Error bars indicate SEM. Asterisks denote a statistical significant decrease (p<0.05) of relative infectivity with respect to particles bearing Gn/Gc. (D) Western blot analysis of the pseudotypes analyzed in panel C employing V5-specific antibody (top) or a VSV M-specific antibody (bottom). Unprocessed glycoprotein precursor proteins are marked with filled triangles while asterisks indicate processing products.

Evidence has been reported that the Gn/Gc proteins of certain bunyaviruses can be processed by signal peptidase during ER import [19–21] and in silico analysis (SignalP 4.0, [33]) also predicted the presence of a signal sequence at the N-terminus of Gc. Moreover, the sequences near the predicted cleavage site in the signal peptide of Gc proteins of other phleboviruses showed high similarity to the corresponding sequences of signal peptides deposited in the MEROPS database (Fig 6A). To investigate the role of the predicted Gc signal peptide in SFTSV-Gn/Gc processing, we deleted the predicted signal peptide or the sequence immediately preceding the predicted cleavage site. Notably, both deletions completely abrogated Gn/Gc processing (Fig 6B) and particle infectivity (Fig 6C) although they were compatible with robust expression and particle incorporation of the unprocessed Gn/Gc precursor (Fig 6C and 6D). Thus, processing of Gn/Gc into mature Gn and Gc proteins depends on the integrity of the predicted signal sequence in Gc and is required for viral infectivity.

## Discussion

Bunyavirus entry into cells is the first essential step in viral replication and the choice of entry factors can constitute a pathogenic molecular signature [34,35]. The M segment of bunyaviruses encodes the proteins required for viral entry into target cells, Gn and Gc but many aspects regarding their biosynthesis and function are poorly understood. The present study focused on Gn and Gc of SFTSV, an emerging bunyavirus. It provides evidence that signal peptidase liberates Gc from the Gn/Gc precursor protein and it demonstrates that both Gn and Gc are required for infectious entry.

SFTSV is a member of the genus *phlebovirus* and is most closely related to the Bhanja serogroup viruses [36]. The M segment of SFTSV is predicted to encode the canonical Gn/Gc protein while an open reading frame for a NSm protein has not been identified. Our previous work indicated that Gc protein is efficiently expressed from an mRNA encoding Gn/Gc [15]. These findings were confirmed in the present study and raised the question how mature Gn and Gc are generated and whether production of the mature proteins is required for viral infectivity.

We first considered the possibility that two RRxR motifs located at positions 5 and 113, respectively, in SFTSV-Gc might constitute target sites for proprotein convertases, which are known to recognize the following consensus motif, (R/K)-2nX-R2 (with n 0–3 amino acids) [37–39]. Such considerations were supported by the well-established role of the proprotein convertase SKI-1 in the generation of Gn of CCHFV [22,23]. However, neither mutation of the RRxR motifs in the context of Gn/Gc nor inhibition of proprotein convertase activity reduced processing of Gn/Gc. Transduction efficiency was diminished upon mutation of the RRxR motifs but this effect was likely due to reduced virion incorporation of the Gc mutants. These results argue against a role of proprotein convertases in the generation of mature Gc. The impact of the methionine at position 1 of Gc on generation of Gc from Gn/Gc encoding mRNA was also examined. A role for the methionine in Gc expression would have been expected if Gn and Gc were separate translation products, with the first codon of Gc serving as a start codon. Such a scenario would apply, for instance, in case of an undetected IRES element in the Gn/Gc mRNA. However, mutation of the Gc methionine did not interfere with production of Gc from mRNA encoding Gn/Gc. These results indicate that proprotein convertase activity, RRxR motifs within Gc as well as the Gc methionine are dispensable for generation of mature Gc from its precursor, Gn/Gc.

We next asked whether signal peptidase might liberate Gc from Gn/Gc. This question was instigated by sequence analysis, which predicted the presence of a signal sequence at the N-terminus of Gc. Notably, the four amino acid residues preceding the predicted cleavage site in SFTSV-Gc (positions -4 to -1) as well as the amino acid residue at position +1 were found to be highly conserved among phleboviruses and residues -4 to -1 matched those most frequently found in signal peptides of cellular proteins. These findings are in line with previous reports documenting a role for signal peptidase in production of the Gc proteins of Uukuniemi virus, RVFV and Hantaan virus [19–21]. The deletion of the predicted Gc signal peptide in the context of SFTSV-Gn/Gc or the removal of the amino acids preceding the predicted cleavage site abrogated Gn/Gc processing, suggesting that signal peptidase might indeed generate the N-terminus of mature Gc. Although formal proof for this conclusion remains to be provided, our data are most compatible with the concept that SFTSV hijacks signal peptidase for liberation of Gc from its precursor, Gn/Gc.

The availability of SFTSV-Gn/Gc mutants allowed us to investigate whether Gn/Gc processing is required for infectivity. Unprocessed Gn/Gc was incorporated into particles but failed to facilitate transduction of target cells, demonstrating that unprocessed SFTSV-Gn/Gc is inactive. This finding is in line with the observation that expression of Gn or Gc alone in particle producing cells was not sufficient for particle infectivity, while particles generated in the presence of both proteins were infectious. This finding raised the question how Gn and Gc contributed to particle infectivity. Based on *in silico* analyses and the X-ray structure of the Rift valley fever virus Gc, bunyavirus Gc proteins were identified as class II membrane fusion proteins.[40]. Therefore, a requirement for SFTSV-Gc for particle infectivity was expected while the contribution of SFTSV-Gn was less clear. The observation that Gn expression was required for particle incorporation of Gc suggested that Gn might function as a chaperon, which facilitates appropriate trafficking of Gc. Indeed, immunofluorescence analysis revealed that Gc was mainly localized in the ER when expressed alone while coexpression of Gn resulted in accumulation of Gc in the Golgi apparatus, the site of bunyavirus assembly and budding. These observations are in keeping with previous studies, which ascribed a chaperon function to the Gn protein of Bunyamwera and Uukuniemi virus [41–43]. One can speculate that a hydrophobic domain in the SFTSV-Gn cytoplasmic tail is responsible for the Golgi localization of the protein, since such a domain is also present in the Gn proteins of other phleboviruses and is known to serve as a Golgi retention signal [44–46]. Moreover, it is conceivable that the long cytoplasmic tail of SFTSV-Gn masks a lysine at position -3 in the cytoplasmic tail of SFTSV-Gc, which is conserved between diverse bunyaviruses and is required for ER localization [46]. The chaperon activity of Gn might depend on Gn-Gc interactions formed with high efficiency only after proteolytic separation of these proteins from the Gn/Gc precursor during glycoprotein biogenesis. Such a scenario might explain why particles generated in Gn/Gc expressing cells incorporated more Gc and were more infectious than their counterparts produced in cells transfected with separate Gn and Gc expression plasmids, despite increased Gn expression and particle incorporation under the latter conditions. However, the role of Gn in SFTSV infection might not be limited to that of a chaperon, since a previous study reported that SFTSV-Gn binds to susceptible cells and interacts with the entry factor non-muscle myosin heavy chain IIA [17]. It is therefore conceivable that SFTSV-Gn facilitates trafficking and maybe folding of Gc and binds to entry factors while Gc drives fusion of viral and cellular membranes, a possibility supported by the recent findings that the Gc proteins of RVFV and SFTSV possess a class II membrane fusion protein architecture [47,48].

In sum, the present study highlights similarities between the biogenesis and biological functions of the Gn and Gc proteins of SFTSV and other bunyaviruses and reports an experimental system which allows the convenient analysis of these proteins.
